# CFTR is a potential marker for nasopharyngeal carcinoma prognosis and metastasis

**DOI:** 10.18632/oncotarget.12762

**Published:** 2016-10-19

**Authors:** Ziwei Tu, Qu Chen, Jie Ting Zhang, Xiaohua Jiang, Yunfei Xia, Hsiao Chang Chan

**Affiliations:** ^1^ Department of Radiation Oncology, Sun Yat-sen University, Cancer Center, Guangzhou, Guangdong, China; ^2^ State Key Laboratory of Oncology in Southern China, Sun Yat-sen University, Guangzhou, Guangdong, China; ^3^ Epithelial Cell Biology Research Center, Key Laboratory for Regenerative Medicine of the Ministry of Education of China, School of Biomedical Sciences, Faculty of Medicine, The Chinese University of Hong Kong, Hong Kong SAR, PR China; ^4^ School of Biomedical Sciences Core Laboratory, Shenzhen Research Institute, The Chinese University of Hong Kong, Shenzhen, PR China; ^5^ Sichuan University-The Chinese University of Hong Kong Joint Laboratory for Reproductive Medicine, West China Second University Hospital, Chengdu, PR China; ^6^ Department of Radiation Oncology, Jiangxi Cancer Hospital, Nanchang, Jiangxi, China

**Keywords:** CFTR, nasopharyngeal carcinoma, prognosis, metastasis

## Abstract

While there is an increasing interest in the correlation of cystic fibrosis transmembrane conductance regulator (CFTR) and cancer incidence, the role of CFTR in nasopharyngeal carcinoma (NPC) development remains unknown. In this study, we aimed to explore the prognostic value of CFTR in NPC patients. The expression of CFTR was determined in NPC cell lines and tissues. Statistical analysis was utilized to evaluate the correlation between CFTR expression levels and clinicopathological characteristics and prognosis in 225 cases of NPC patients. The results showed that CFTR was down-regulated in NPC tissues and cell lines. Low expression of CFTR was correlated with advanced stage (*p* = 0.026), distant metastasis (*p* < 0.001) and poor prognosis (*p* < 0.01). Multivariate analysis identified CFTR as an independent prognostic factor (*p* = 0.003). Additionally, wound healing and transwell assays revealed that overexpression of CFTR inhibited NPC cell migration and invasion, whereas knockdown of CFTR promoted cell migration and invasion. Thus, the current study indicates that CFTR, as demonstrated to play an important role in tumor migration and invasion, may be used as a potential prognostic indicator in NPC.

## INTRODUCTION

Nasopharyngeal carcinoma (NPC) is the most common cancer originating in the nasopharynx. The incidence of NPC has remained high in southeast Asia, particularly in southern China (~25–30 per 100,000 persons per year) [1]. Owing to advances in precise radiotherapy and comprehensive chemotherapy, localregional control and survival of primary NPC patients have been improving significantly. Nonetheless, more than 30% of patients will relapse with either localregional recurrence or distant metastases [2], and the overall survival rate of recurrent patients is poor with median survival ranging from 7.2 to 22 months [3–5]. The majority of cancer death is attributed to distant metastasis, which is a predominant reason of treatment failure in NPC patients who do not present metastases at diagnosis. Hence, better understanding of the mechanisms underlying the acquisition of the invasive phenotype, and development of novel prognostic indicators are important for NPC treatment.

Cystic fibrosis transmembrane conductance regulator (CFTR) is a cAMP-activated chloride channel, mutation of which results in cystic fibrosis (CF), a common fatal autosomal recessive disease [6, 7]. Since treatment strategies for CF patients have been improved greatly, the life span of CF patients is prolonged significantly. In turn, there has been recent interest in the risk of various cancers in CF patients and carriers of CFTR mutations [8–13]. Disruption of CFTR function and/or dysregulation of CFTR expression have been associated with a wide range of cancers including esophageal, breast, gastric, hepatobiliary, gall bladder, prostate, lung, small intestine and colorectal cancers (CRC) [11, 14–22]. Furthermore, down-regulation of CFTR has been correlated with cancer progression, and proposed to be a prognostic predictor for lung cancer, breast cancer and colon cancer [19–22]. However, high levels of CFTR expression have also been reported to be associated with invasive phenotype and poor prognosis in cervical and ovarian cancers [23–25]. The seemingly contradictory findings indicate the role of CFTR in cancer development might be tissue specific. Of note, the role of CFTR in NPC progression, and its prognostic significance and impact on NPC patient survival have never been explored.

In the present study, we determined the expression of CFTR in NPC cell lines and tissue samples, and evaluated its correlation with clinical characteristics and patient prognosis. Our results show that low expression levels of CFTR are associated with cancer progression and poor survival of NPC patients. We also demonstrate that CFTR manipulation in NPC cell lines affects cell migration and invasion, providing mechanistic basis for the role of CFTR in NPC development.

## RESULTS

### CFTR expression is down-regulated in NPC cell lines and tissues

**Figure 1:**
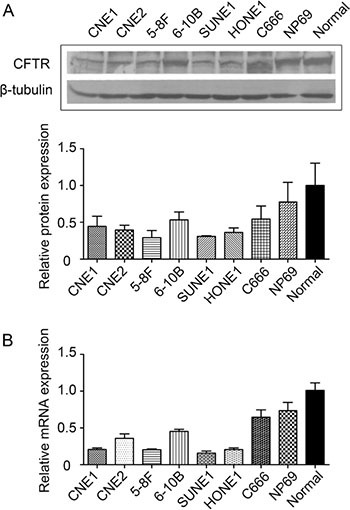
Expression of CFTR in NPC cell lines and normal nasopharyngeal epithelial cell lines (**A**) Western blotting analysis of CFTR protein in normal nasopharyngeal epithelial cells, immortalized nasopharyngeal epithelial cell line (NP69) and NPC cell lines (CNE1, CNE2, 5–8F, 6–10B, SUNE1, HONE1 and C666). (**B**) Real-time PCR analysis of CFTR mRNA in the same cell lines as described in A. Quantification analysis of data is expressed as the Mean ± SEM from three independent experiments.

We first determined the expression levels of CFTR in various NPC cell lines compared to the immortalized nasopharyngeal epithelial cells (NP69) and normal nasopharyngeal epithelial cells (Normal). Our western blotting analysis revealed that CFTR expression levels were lower in all NPC cell lines than that in NP69 and Normal cells. Of note, CFTR expression was lower in high-metastasis 5–8F cells than that in low-metastasis 6–10B cells, which are originated from the same SUNE-1 cell line [26] (Figure 1A). Consistent with the protein expression, decreased expression of CFTR mRNA was observed in all NPC cell lines examined except for C666 cells (Figure 1B). Thus, the expression levels of CFTR are downregulated in NPC cell lines compared to that in normal and immortalized cells.

**Figure 2:**
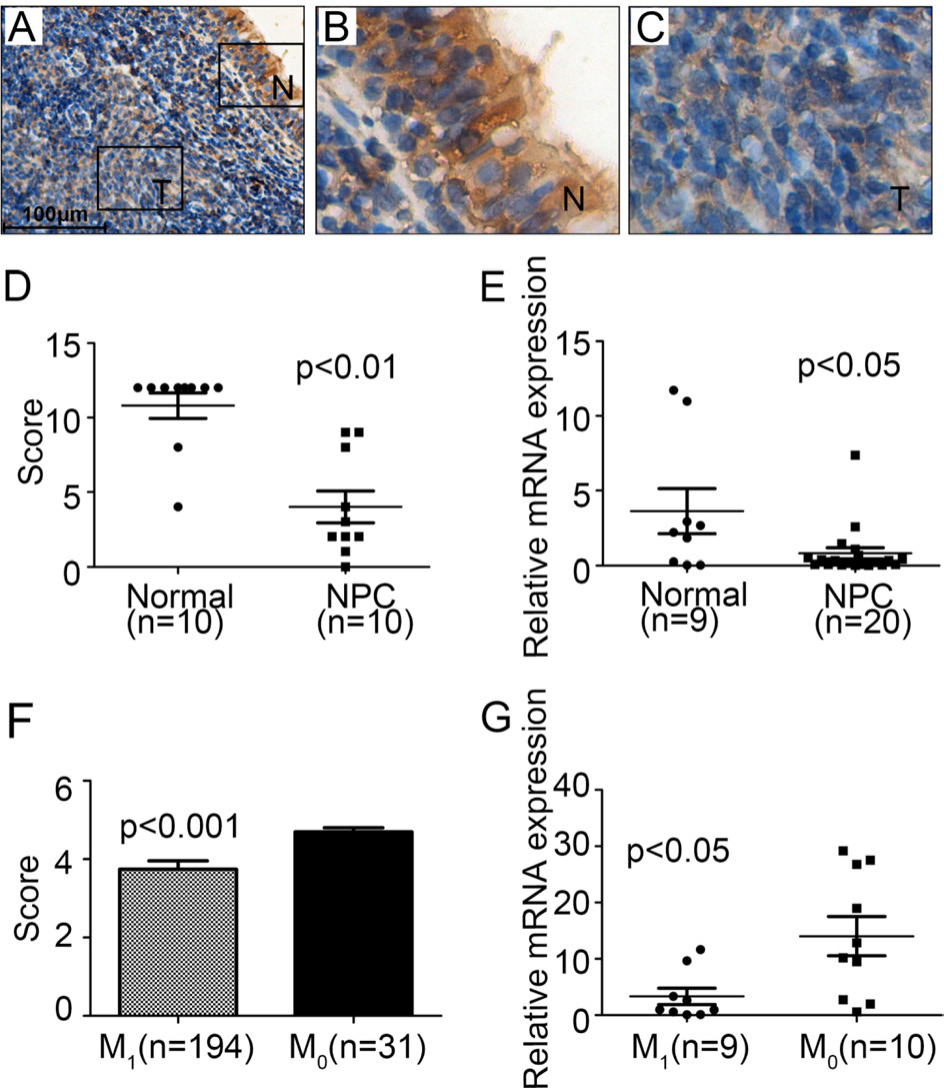
Low expression levels of CFTR in NPC tissues (**A**–**C**) Immunohistochemical staining of cell nuclei (blue) and CFTR protein (brown) in representative images from NPC tumor tissue (*n* = 10). It can be seen that CFTR is mainly expressed at the cytoplasm of nasopharyngeal epithelial cells. Compared to adjacent normal tissue, CFTR expression is dramatically decreased in tumor tissue. Squared area captured at A is enlarged in B and C. scale bar: 100 μm. (**D**) Immunohistochemistry staining of CFTR expression in normal (*n* = 10) and NPC tissues (*n* = 10). The expression of CFTR is significantly decreased in NPC patient samples, *p* < 0.01. (**E**) Real-time PCR analysis of CFTR mRNA expression in normal nasopharyngeal biopsies (Normal, *n* = 9) and nasopharyngeal carcinoma biopsies (NPC, *n* = 20), *p* < 0.05. (**F**) IHC score of CFTR expression in NPC patients with (M_1_, *n* = 194) or without (M_0_, *n* = 31) metastasis, *p* < 0.001. (**G**) Real-time PCR analysis of CFTR mRNA in NPC patients with (M_1_, *n* = 9) or without(M_0_, *n* = 10) metastasis, *p* < 0.05.

We proceed to determine the expression and localization of CFTR in primary NPC tissues. In one of the NPC sections containing both tumor tissue and adjacent normal tissue, we observed a transitional expression pattern of CFTR with strong expression in the normal nasopharyngeal tissue but much decreased expression in tumor region (Figure 2A–2C). Thus, we further evaluated the expression levels of CFTR by immunochemistry in 10 cases of NPC samples and 10 normal tissues. Our results showed that the expression levels of CFTR were much lower in NPC tissues compared with that in nasopharyngeal epithelia (*p* < 0.01) (Figure 2D). To further quantify the expression levels of CFTR, we examined the expression of CFTR in 9 normal tissue samples and 20 NPC tissue samples using real-time RT-PCR analysis. CFTR was found to be significantly downregulated in NPC tissue samples compared to that in normal samples (*p* < 0.05) (Figure 2E). These data suggest that CFTR expression is downregulated in NPC samples.

### Low CFTR expression is associated with advanced disease in NPC

Next, we attempted to evaluate the correlation of CFTR expression with NPC progression. We used another cohort of 225 paraffin-embedded NPC specimens diagnosed between 1994 and 1999 to further examine the expression of CFTR protein by immunohistochemical staining. We first evaluated CFTR expression levels in accordance with patients' metastasis status. Statistical analysis revealed that CFTR expression of patients with metastasis (*n* = 194) was significantly lower than patients without metastasis (*n* = 31, *p* < 0.001) (Figure 2F). Further analysis of CFTR mRNA levels according to patients' metastasis status showed that CFTR expression in patients with metastasis (*n* = 9) was significantly lower than patients without metastasis (*n* = 10, *p* < 0.05) (Figure 2G).

**Figure 3:**
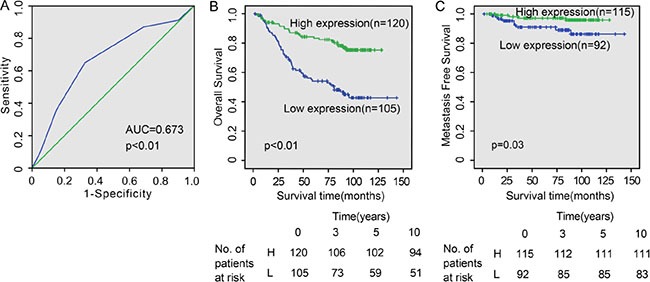
CFTR expression levels correlate with NPC patient survival (**A**) The receiver operating characteristic (ROC) curve of CFTR expression for predicting survival of NPC patients. (**B** and **C**) Kaplan-Meier analysis for patients with different CFTR expression levels. Low expression of CFTR is closely correlated with poor overall survival (*p* < 0.01) (B) and metastasis-free survival (*p* = 0.03) (C).

To further investigate the association of CFTR expression levels with NPC progression, we determined the best cutoff expression level using ROC curve in the test set (*n* = 225). The CFTR expression cutoff value was determined to be 4.5 with 64.8% sensitivity and 67.5% specificity (Figure 3A). We thus divided the cohort into high expression (score > 4.5) and low expression (score ≤ 4.5) populations based on the cutoff value. CFTR levels were statistically analyzed to identify an association with the clinicopathologic characteristics of NPC. As shown in Table 1, CFTR expression was significantly correlated with clinical stage (*p* = 0.026) and distant metastasis (*p* = 0.003). Nevertheless, there was no significant correlation between CFTR expression and gender, age, histological classification, T classification, N classification, relapse and skull-base invasion.

**Table 1:** Correlation between the clinicopathologic features and expression of CFTR

Characteristics	*N*	CFTR		χ^2^	*P* values
		Low expression	High expression		
Gender					
Male	171	80	91	0.004	0.95
Female	54	25	29		
Age					
< 45	109	48	61	0.588	0.443
≥ 45	116	57	59		
Histological classification					
Type II	11	6	5	0.288	0.591
Type III	214	99	115		
Clinical stage					
I-II	97	37	60	4.976	0.026*
III-IV	128	68	60		
T					
T1–T2	145	61	84	3.464	0.063
T3–T4	80	44	36		
N					
N0	137	59	78	1.825	0.177
N1–N3	88	46	42		
M					
M0	194	83	111	8.531	0.003*
M1	31	22	9		
Relapse					
Yes	201	94	107	0.007	0.931
No	24	11	13		
Skull-based invasion					
Yes	177	77	100	3.337	0.068
No	48	28	20		
Radiotherapy response					
Sensitive	187	83	104	2.316	0.128
Resistant	38	22	16		

* Significantly different.

### Lower CFTR expression is correlated with poor prognosis and inferior survival in NPC

Since metastasis is the main cause of tumor relapse and high mortality of NPC, we also evaluated the prognostic potential of CFTR using clinical outcomes collected by the follow-up study. The median follow-up time for the 225 NPC patients was 83.9 months, ranging from 1.8 to 143.1 months. Through Kaplan-Meier survival analysis, patients with high expression levels (higher than 4.5, *n* = 120) of CFTR had longer overall survival than patients with lower CFTR expression levels (lower than 4.5, *n* = 105) (*p* < 0.01) (Figure 3B). Among them, patients with higher CFTR levels had better survival status, presenting longer metastasis free time compared to those with poor survival (*p* = 0.03) (Figure 3C). In addition, it should be noted that patients with high CFTR levels had higher 10-year survival rate (41.7%), compared to those with lower CFTR levels (22.6%) (Figure 3B) Thus, lower expression of CFTR is significantly associated with disease progression and poor prognosis in NPC.

Univariate analysis indicated that apart from CFTR expression levels (*p* < 0.001), gender (*p* = 0.037), histological classification (*p* = 0.015), T classification (*p* < 0.001), N classification (*p* = 0.001), distant metastasis (*p* < 0.001), relapse (*p* = 0.003), skull-based invasion (*p* = 0.003) and radiotherapy response (*p* < 0.001) were also significantly correlated with patient survival (Table 2, left panel). Multivariate analysis showed that T classification (*p* = 0.036), N classification (*p* = 0.003), distant metastasis (*p* < 0.001), relapse (*p* = 0.002) and CFTR expression level (*p* = 0.003) were independent prognostic factors for NPC (Table 2, right panel). Thus, our findings indicate that CFTR expression level, as an independent prognostic factor, is associated with clinical prognosis of NPC patients.

**Table 2:** Univariate and multivariate analysis of factors associated with overall survival

Variables	Univariate analysis			Multivariate analysis		
	HR	95% CI	*P*	HR	95% CI	*P*
Gender Male *vs* Female	1.923	1.041–3.553	**0.037**			NS
Age(years) < 45 *vs* ≥ 45	0.736	0.473–1.147	0.176			
Histological classification Type II *vs* Type III	0.381	0.175–0.828	**0.015**			NS
T classification T1–T2 *vs* T3–T4	0.448	0.288–0.695	**< 0.001**	0.528	0.29–0.960	**0.036**
N classification N0 *vs* N1–N3	0.487	0.314–0.756	**0.001**	0.452	0.267–0.768	**0.003**
Distant metastasis No *vs* Yes	0.181	0.110–0.297	**< 0.001**	0.295	0.165–0.526	**< 0.001**
Relapse No *vs* Yes	0.43	0.245–0.756	**0.003**	0.404	0.225–0.726	**0.002**
Skull-based invasion No *vs* Yes	0.477	0.295–0.772	**0.003**			NS
Radiotherapy response Sensitive *vs* Resistant	0.407	0.249–0.665	**< 0.001**			NS
CFTR Low *vs* High	3.016	1.886–4.824	**< 0.001**	2.126	1.286–3.516	**0.003**

NS, not significant.

### CFTR affects migration and invasion abilities of NPC cell lines

**Figure 4:**
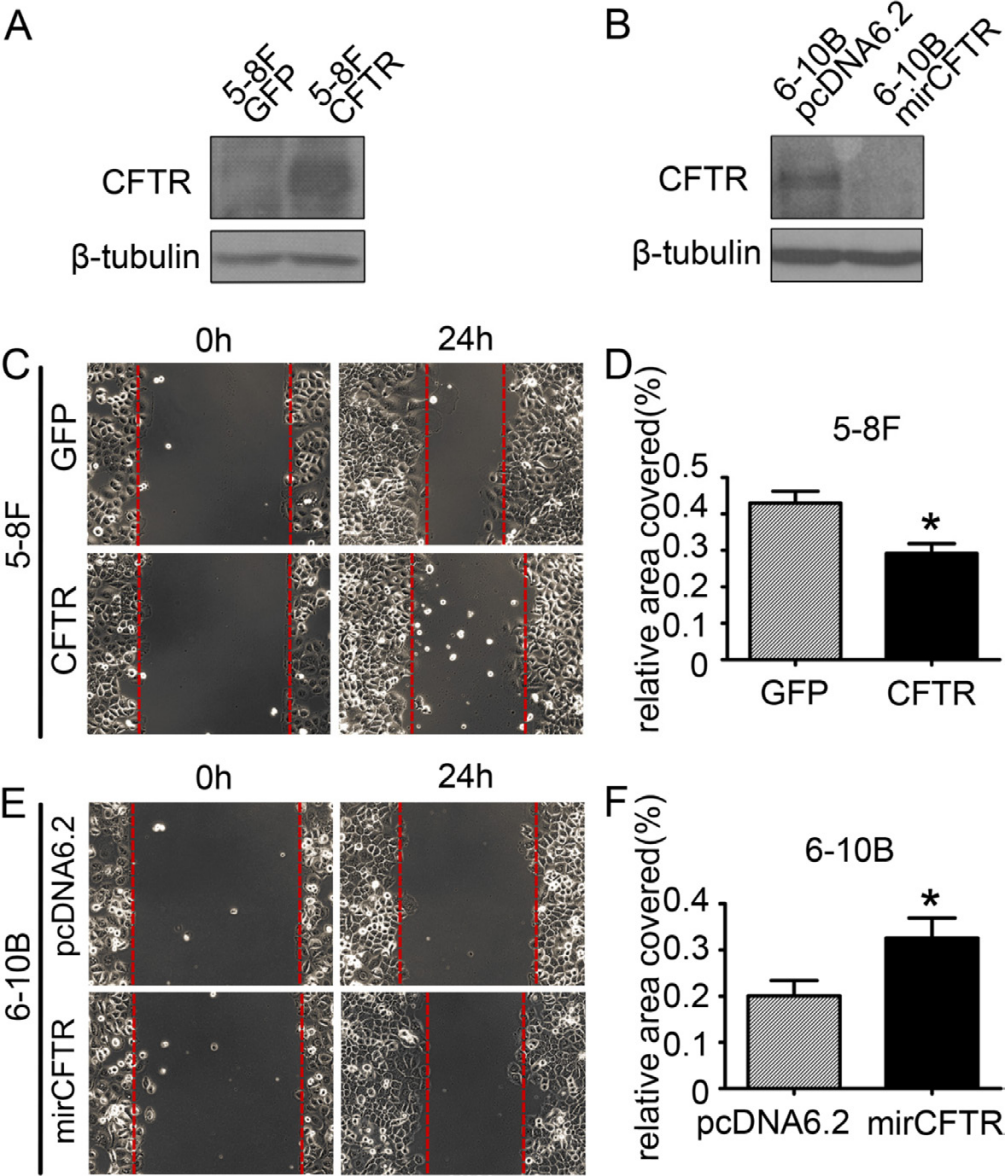
CFTR affects NPC cell migration (**A**) The expression of CFTR in control and CFTR-overexpressing 5–8F cells as determined by western blotting. (**B**) The expression of CFTR in control and CFTR-knocking-down 6–10B cells as determined by western blotting. (**C**) Overexpression of CFTR inhibits cell migration in 5–8F cells as demonstrated by wound healing assays. (**D**) Quantification analysis of cell migration in 5–8F cells is expressed as the Mean ± SEM from three independent experiments (*p < 0.05). (**E**) Knockdown of CFTR expression promotes cell migration in 6–10B cells. (**F**) Quantification analysis of cell migration in 6–10B cells is expressed as the Mean ± SEM from three independent experiments (*p < 0.05).

**Figure 5:**
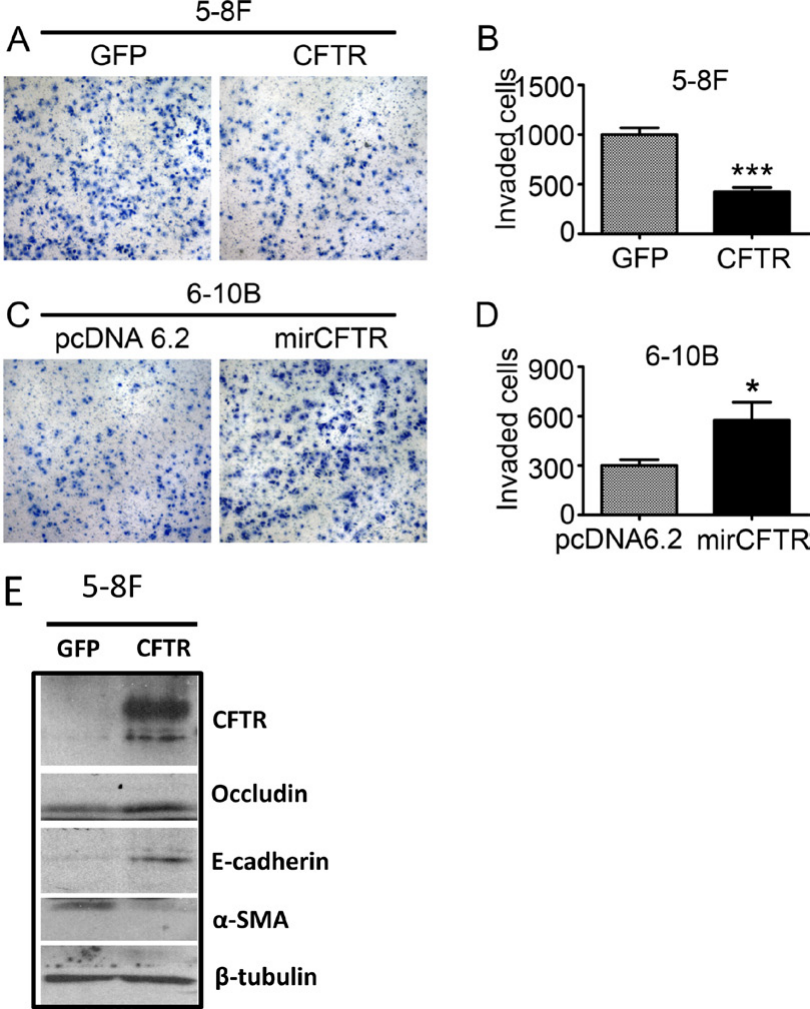
CFTR regulates NPC cell invasion (**A** and **B**) Overexpression of CFTR inhibits cell invasion in 5–8F cells as demonstrated by transwell assays. Quantification analysis of data is expressed as the Mean ± SEM from three independent experiments (***p < 0.001). (**C** and **D**) Knockdown of CFTR expression promotes cell invasion in 6–10B cells. Quantification analysis of data is expressed as the Mean ± SEM from three independent experiments (*p < 0.05). (**E**) The expression of EMT markers was determined by western blotting in control and CFTR-overexpressing 5–8F cells.

**Figure 6:**
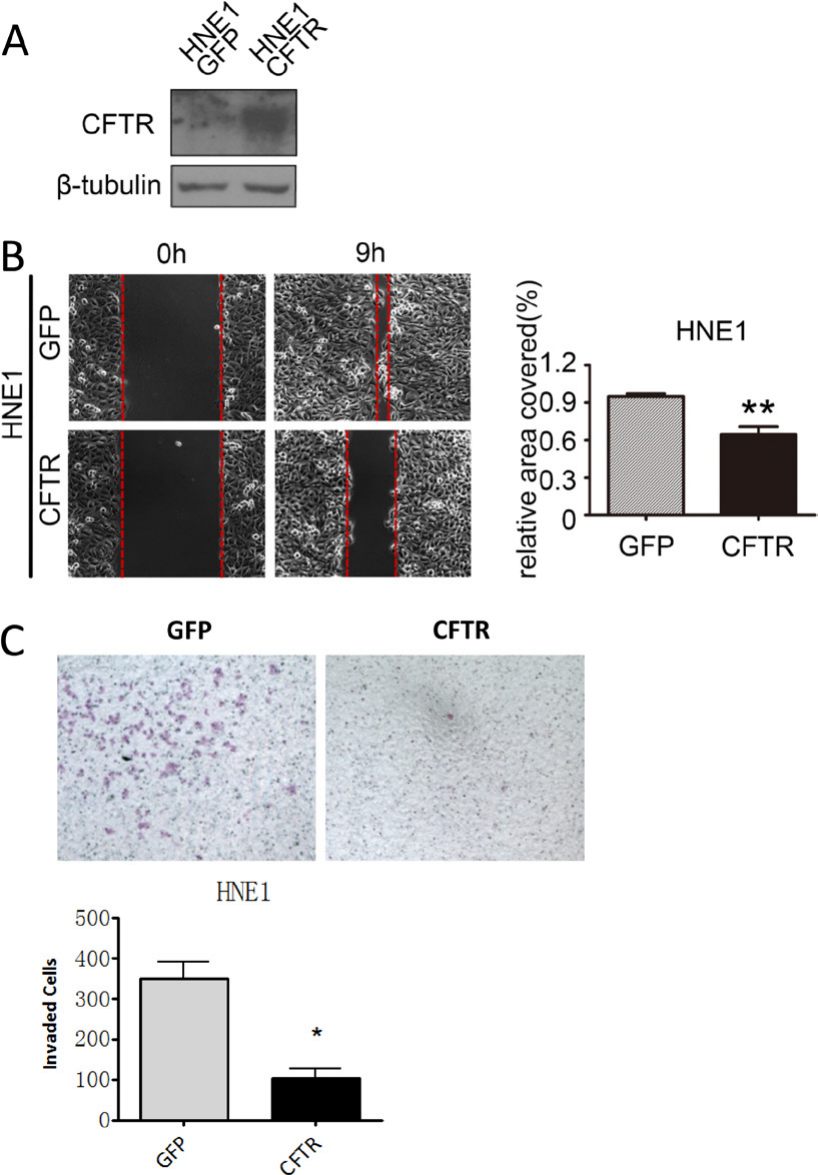
CFTR regulates cell migration and invasion in HNE1 cells (**A**) The expression of CFTR in control and CFTR-overexpressing HNE1 cells as determined by western blotting. (**B**) Overexpression of CFTR inhibits cell migration in HNE1 cells as demonstrated by wound healing assays. Quantification analysis of data is expressed as the Mean ± SEM from three independent experiments (**p < 0.01). (**C**) Overexpression of CFTR inhibits cell invasion in HNE1 cells as demonstrated by transwell assays. Quantification analysis of data is expressed as the Mean ± SEM from three independent experiments (*p < 0.05).

The observed association between CFTR expression levels and NPC metastasis and prognosis prompted us to investigate whether CFTR gene manipulation might affect the migration and/ or invasion of NPC cells. To perform the cell functional study in comparable cell lines, we used 5–8F and 6–10B cells which are two subclones of SUNE-1 with high-metastatic and low- metastatic tendency respectively. Thus, they are good models for investigating the role of CFTR in metastasis of NPC. We exogenously overexpressed CFTR in 5–8F cells, and knocked down CFTR expression in 6–10B cells. The transfection efficiency of CFTR was confirmed by western blotting (Figure 4A and 4B). Our results showed that overexpression of CFTR in 5–8F cells strongly inhibited cell migration (Figure 4C and 4D, *p* < 0.05), whereas knockdown of CFTR in 6–10B cells significantly promoted cell migration (Figure 4E and 4F, *p* < 0.05). We further determined the effect of CFTR on cell invasion by transwell assay. As shown in Figure 5A–5D, numbers of invasive cells were dramatically decreased in CFTR overexpressing 5–8Fcells (Figure 5A and 5B, *p* < 0.001). On the contrary, numbers of invasive cells increased significantly in CFTR knockdown 6–10B cells (Figure 5C and 5D, *p* < 0.05). The role of CFTR in NPC cell migration and invasion was validated in another NPC cell line HNE1, as overexpression of CFTR in HNE1 significantly suppressed cell migration and invasion (Figure 6). As a first step to investigate the mechanistic role of CFTR in cell migration and invasion, we determined the expression of epithelial-mesenchymal transition (EMT) markers in CFTR overexpressing 5–8F cells compared to their control. Our results showed that overexpression of CFTR increased the expression of epithelial markers Occludin and E-cadherin, whereas decreased the expression of mesenchymal marker SMA (Figure 5E), indicating CFTR might regulate EMT process in NPC cell lines. Taken together, these data indicate that CFTR plays critical role in the regulation of invasive phenotype of NPC.

## DISCUSSION

While metastasis has been the major cause of treatment failure and death of NPC patients [27], the molecular mechanisms underlying NPC metastasis are still largely unknown. Hence, reliable biomarkers for predicting metastasis and patient prognosis are still lacking for NPC patients. In the present study, we found that CFTR expression was significantly down-regulated in NPC cell lines and tissues. By analyzing the CFTR expression levels against clinicopathologic factors of NPC patients, this study, for the first time, has revealed that low expression level of CFTR is significantly correlated with advanced disease and poor prognosis of NPC patients. These data reveal a previously undefined role of CFTR in NPC development.

We have first examined the expression of CFTR in NPC cell lines, and found that the expression levels of CFTR are globally downregulated in NPC cell lines compared to normal nasal epithelial cells (Figure 1A and 1B). It is noteworthy that the expression of CFTR in C666 cells with persistent EBV infection is comparable to that in normal nasal epithelia. Interestingly, previous studies showed that the enhanced proteasomal degradation of CFTR-associated ligand (CAL) in Golgi mediated by the specific interaction with HPV 16/18 E6 domain led to CFTR overexpression in the plasma membrane [28, 29]. In addition, CFTR expression was also reported to be associated with HPV infection in cervical carcinoma [25]. Thus, it is plausible that EBV infection may induce abnormal expression of CFTR in C666 cells.

We determined the expression of CFTR in two cohorts of NPC samples and correlated it with NPC clinicopathologic characteristics and survival rate. Our results show that the reduced expression of CFTR is correlated with advanced disease stage and distant metastasis, but not tumor size or lymph node metastasis, indicating low CFTR expression is related to more advanced disease. Since metastasis is the main cause of tumor relapse and high mortality of NPC, we also evaluated the prognostic potential of CFTR using clinical outcomes. Statistical analysis shows that low CFTR expression is correlated with shorter survival of NPC patients (Tables 1 and 2, Figure 3). Collectively, these results clearly indicate that low level of CFTR expression is indicative of advanced disease and poor prognosis in NPC. Previous studies from both our groups and others have also shown the correlation of CFTR expression levels and cancer prognosis in different cancers [18–24]. Consistent with the finding in NPC in the present study, we have previously reported that low CFTR expression is correlated with cancer progression and poor prognosis in prostate, breast, colon and lung cancers [18–20, 23]. In contrast, CFTR was found to be highly expressed in cervical cancer and associated with poor prognosis [25]. These results suggest that CFTR may play different roles in different cell types and thus different cancers. Therefore, it is clinically important to study CFTR in each cancer type to determine its prognostic potential.

Emerging evidence has indicated the role of CFTR in caner EMT and metastasis [18–20]. In the present study, we have shown that overexpression of CFTR suppresses NPC cell migration and invasion, whereas knockdown of CFTR promotes them (Figures 4–6). These results are in line with the observed changes in NPC samples, and consistent with a metastasis-suppressing role of CFTR. Our previous studies have indicated that dysfunction of CFTR promotes EMT and cancer metastasis via both genetic and epigenetic pathways, such as uPA, NF-κB, MAPK and miR-193 [18–20]. Interestingly, in this study, we have also found that overexpression of CFTR upregulates epithelial markers whereas downregulates mesenchymal marker, indicating EMT process may play a role in mediating the metastasis-suppressing role of CFTR in NPC. In addition, as the ABC family protein, CFTR and multidrug resistance protein MRP (multi-drug resistant protein) can interact with each other [30–34]. Thus, CFTR may be associated with tumor drug resistance, modulating the efficacy of chemotherapy and then affecting patient metastasis rate after chemotherapy. The exact mechanisms for the effect of CFTR on nasopharyngeal carcinoma metastasis and prognosis warrant further investigation.

In summary, our results indicate that CFTR expression is down-regulated in NPC, and low protein level of CFTR is associated with poor prognosis. Thus, CFTR could be a novel and useful prognostic marker for NPC patients. However, the possible underlying mechanisms for CFTR modulating tumor progression remain to be elucidated, which might eventually lead to the development of new anti-NPC strategies.

## MATERIALS AND METHODS

### Cell culture and transfection

Cell lines were obtained from Sun Yat-Sen University Cancer Center [35, 36]. NP69 cells and primary nasopharyngeal epithelial cells were grown in keratinocyte/serum-free (KSF) medium (Invitrogen), other cell lines were cultured in RPMI 1640 (Invitrogen, Carlsbad, CA) supplemented with 10% fetal bovine serum (FBS; Hyclone, Logan, UT), penicillin (100 units/ml), and streptomycin (100 units/ml) in a humidified 5% CO_2_incubator at 37°C.

The 5–8F cells and HNE1 cell lines were transfected with 2.5 μg pEGFPC3 plasmid expressing wild-type CFTR (kindly provided by Professor Tzyh-Chang Hwang, University of Missouri-Columbia) and 5 μl Lipofectamine 2000 (Invitrogen, Camarillo CA), and selected in full medium containing G418 (Calbiochem, Schwalbach, Germany) at 400 μg/ml. In contrast, 6–10B cells were transfected with pcDNA6.2-miR-CFTR or pcDNA6.2-miR-lacz (Lift Technologies), and the stably-transfected cell lines were obtained by selection for Blasticidin resistance (2.5 μg/ml) [18].

### Wound-healing assay

NPC cells were suspended and seeded in 6-well plates (1 × 10^6^ cells/well), and replaced the culture medium with FBS-free 1640 before scraping a wound across the cell monolayer with pipette tips. The restoration of the wound was tracked and recorded by a real-time live cell imaging microscope system (Carl Zeiss, Oberkochen, Germany) at 1 hour interval for 24 hours. Cell migration ability alteration was determined by comparing reduced areas of the scratches.

### Cell invasion assay

Invasion assay was performed with transwell chamber (Corning Incorporated, MA, USA) pre-coated with 500 μg/ml Matrigel. Cells were seeded to the upper chamber at 20,000 cells/well and incubation for 48 h. Cells that invaded through the membrane of transwells were fixed in 4% paraformaldehyde for 20 min and stained with 0.5% crystal violet solution for 30 min. The number of invaded cells was counted under a microscope.

### Western blotting

Western blot analysis was performed as described previously [37]. Briefly, Cells were washed three times with cold PBS and total cellular proteins were extracted with lysis buffer. The protein concentration was detected by BCA Protein Assay Kit (Beyotime Biotechnology). Equal amounts of protein samples was subjected to 8% SDS-PAGE gel for electrophoresis and transferred to polyvinylidene fluoride (PVDF) membranes (Millipore corporation, USA). The membrane was incubated with primary antibody 4°C overnight and HRP-conjugated secondary antibody. The protein bands were visualized by enhanced chemiluminescence (Amersham Pharmacia Biotech, Piscataway, NJ) following the manufacturer's instructions. Antibodies: CFTR (1:200; Almone Lab; ACL-006), β-tubulin (1:2000; Santa Cruz; sc-9104).

### Real time quantitative PCR

Total RNA of NPC cells was extracted using TRIzol reagent (Invitrogen Corporation, NY, USA) according to the manufacturer's suggested protocols. Subsequently, the first-strand complementary DNA (cDNA) was synthesized with 3 μg total RNA. Real time-PCR was conducted with an Applied Biosystems 7500 Fast Real-Time PCR System. For normalization, GAPDH was used as endogenous control. The primer sequences are sense 5′- TGC CCT TCG GCG ATG TTT -3′ and antisense 5′- GCG ATA GAG CGT TCC TCC TTG -3′ for CFTR, and sense 5′- CTC CTC CTG TTC GAC AGT CAG C -3′, antisense 5′- CCC AAT ACG ACC AAA TCC GTT -3′ for GAPDH.

### Tissue samples

All tissue samples used in this study were acquired from Sun Yat-Sen University Cancer Center. The diagnosis of NPC was confirmed at the time of original diagnosis and the presence of tumor cells was verified by a consultant pathologist using H&E staining of frozen sections. All human specimens and correlative data were obtained following protocol reviewed and approved by the local Ethical Committee and all patients gave their written informed consent. 225 paraffin-embedded NPC tissue specimens were diagnosed between 1994 and 1999, the case selection criteria: 1) initial diagnosis of NPC; 2) age 20–75 years; 3) stageI-IV; 4) availability of tumor tissue and follow-up information. Another 10 paraffin-embedded NPC slides and 10 normal slides were diagnosed between 2011 and 2013. Freshly frozen tissue samples of 39 nasopharyngeal carcinoma biopsies and 9 noncancerous nasopharyngeal biopsies from the Department of Radiation Oncology were also included.

Clinical follow-up was acquired from the respective patient physicians and through review of medical records. The follow-up period was defined as the interval from the date of diagnosis to the date of death or the last follow-up. Patients enrolled were followed-up at least every 3 months during the first 2 years and then every 6 months thereafter. Median follow-up was 83.9months (range, 1.8–143.1 months). For survival analysis, metastasis-free survival was defined as the minimum interval from the date of diagnosis to the date of tumor recurrence and occurrence of a second malignancy, death, or last follow-up. Overall survival (OS) was defined as the interval from the date of diagnosis to the date of death or last follow-up. Patients alive with local recurrence or metastatic were considered as in disease survival.

### Immunohistochemical staining (IHC)

IHC analysis of CFTR was conducted according to a previously described method [37]. Briefly, the paraffin-embedded tissue sections were baked for 2 h at 65°C, dewaxed with xylenes and then rehydrated with graded ethanol to distilled water. The sections were boiled in EDTA antigen retrieval buffer (pH 8.0) in a microwave oven for antigen retrieval. After being treated with 0.3% H_2_O_2_ and normal goat serum, the slides were incubated at 4°C with a CFTR antibody (1:100; Almone Lab; ACL-006) overnight. Tissue sections were then washed with PBST, and incubated with a biotinylated anti-rabbit secondary antibody. Subsequently, the slides were incubated with streptavidin horseradish peroxidase complex at 37°C for 30 min, and finally developed using diaminobenzidine tetrahydrochloride (DAB).

### Scoring of IHC staining results

CFTR expression was scored visually by two well-trained independent pathologists in Sun Yat-sen University Cancer Center. The entire tissue section was scored by the intensity and extent of the staining (the percentages of the positive staining areas in relation to the whole carcinoma area or the entire section for the normal samples). The staining intensity scores were determined as 0 (no staining), 1 (weak staining exhibited as light yellow), 2 (moderate staining exhibited as yellow brown), or 3 (strong staining exhibited as brown). The extent of staining scores were determined as 0 (0%), 1 (1 to 25%), 2 (26 to 50%), 3 (51 to75%), or 4 (76 to 100%). The final immunoreactive score was determined by adding the intensity scores with the extent of positivity scores of stained cells, with the minimum score of 0 and a maximum score of 7.

### Statistical analyses

ROC curve analysis was employed to determine the cutoff value for expression of CFTR. The correlation between CFTR expression and the clinicopathologic features of the NPC patients was analyzed by a χ^2^-test. Survival curves were obtained with the Kaplan-Meier method (version 11; SPSS. Chicago, IL, USA). Log-rank test was used to compare differences between survival curves and differences were considered to be statistically significant at *p* < 0.05 [38].
